# Palbociclib as an early-line treatment for Japanese patients with hormone receptor–positive/human epidermal growth factor receptor 2–negative advanced breast cancer: a review of clinical trial and real-world data

**DOI:** 10.1007/s10147-021-02013-8

**Published:** 2021-10-26

**Authors:** Norikazu Masuda, Nobuyoshi Kosaka, Hiroji Iwata, Masakazu Toi

**Affiliations:** 1 National Hospital Organization Osaka National Hospital, 2-1-14 Hoenzaka, Chuou-ku, Osaka-city, 540-0006 Japan; 2 Pfizer Japan Inc, Tokyo, Japan; 3 Aichi Cancer Center Hospital, Aichi, Japan; 4 Graduate School of Medicine, Kyoto University, Kyoto, Japan

**Keywords:** Advanced breast cancer, Clinical trial, HR+/HER2–, Palbociclib, Real-world

## Abstract

Breast cancer is the most common type of cancer among women worldwide and in Japan. The majority of breast cancers are hormone receptor–positive (HR+)/human epidermal growth factor receptor 2–negative (HER2‒), and endocrine therapy is an effective therapy for this type of breast cancer. However, recent substantial advances have been made in the management of HR+/HER2‒ advanced breast cancer (ABC) with the advent of targeted therapies, such as cyclin-dependent kinase 4/6 (CDK4/6) inhibitors, resulting in significant improvements in survival outcomes versus endocrine therapy alone. To evaluate the optimal use of palbociclib, a CDK4/6 inhibitor, in HR+/HER2– ABC, this review summarizes clinical trial and real-world data for palbociclib. In addition, current biomarker studies in palbociclib clinical research are reviewed. In Japanese patients, palbociclib was shown to be effective with a manageable safety profile, although differences were observed in the frequency of adverse event and dosing parameters. Current evidence supporting palbociclib as a first-line treatment strategy for patients with HR+/HER2‒ ABC in Asia, and specifically japan, is also discussed.

## Introduction

Breast cancer is the most common cancer among women worldwide and also in Japan [1] and is the fifth leading cause of cancer-related death in Japanese women [2]. In eastern Asia (inclusive of Japan) in 2018, the incidence of breast cancer was 39.2 per 100,000 females, whereas the mortality rate was 8.6 per 100,000 females [1]. Four main molecular subtypes of breast cancer exist (i.e., hormone receptor–positive/human epidermal growth factor receptor 2–negative [HR+/HER2–], HR+/HER2+ , HR‒/HER2‒, and HR‒/HER2+) [3]. Among patients diagnosed with primary breast cancer, the majority (71–73%) of breast cancers are HR+/HER2‒ [3, 4].

Monotherapy with an aromatase inhibitor or fulvestrant is a reasonable treatment option for HR+ advanced breast cancer (ABC) considering the economic and clinical benefit to patients. A previous study in patients with estrogen receptor–positive (ER+) ABC or metastatic breast cancer (MBC) reported a median overall survival (OS) of 54.1 months with fulvestrant and 48.4 months with anastrozole [5]. In Japanese patients with HR+ ABC who were treated with anastrozole, the median time to progression was 13.7 months, and median OS was 60.1 months [6]. However, it is essential to consider how OS can be extended beyond 5 years in patients with ABC, since data suggest that initial treatment of ABC with chemotherapy does not provide a survival advantage over endocrine therapy [7].

**Fig. 1:**
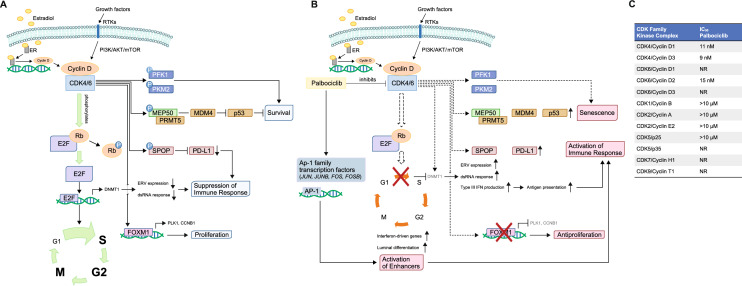
Mechanism of action of palbociclib [8, 9, 30, 70–77]. Panel **A** shows how the CDK4/6:cyclin D1 complex phosphorylates not only the retinoblastoma protein, which releases the E2F transcription factor, driving progression from the G_1_ to the S phase of the cell cycle, but also FOXM1 (activates the expression of other cell-cycle genes), MEP50 (p53 signaling pathway), PFK1 and PKM2 (glycolytic enzymes), and SPOP (ubiquitin ligase subunit) that helps with PD-L1 degradation [9, 70, 75]. CDKs and cyclins have been shown to be dysregulated in breast cancer cells [71]. However, studies have shown that inhibition of CDK4/6 activity (e.g., with palbociclib) halts cell-cycle progression and prevents tumor cell division (Panel **B**) [30]. Furthermore, inhibition of CDK4/6:cyclin D1 activity by palbociclib may activate an immune response by promoting immune-related gene expression through activation of enhancers, PD-L1 expression, or antigen presentation [75, 77]. Thus, drugs that inhibit CDK4/6:cyclin D1 activity became a focus of breast cancer treatment. Findings from a preclinical study evaluating the growth inhibitory effects of a CDK4/6 inhibitor across a panel of molecularly characterized breast cancer cell lines identified the most potent activity in cell lines that were estrogen receptor (ER)‒positive (ER+) and HER2‒ amplified [74]. Panel C shows the binding capacity of palbociclib to each CDK complex (IC_50_ by cell-free assay) [78]. Dashed lines represent outcomes after CDK4/6:cyclin D1 inhibition. *AP-1* activator protein-1; *CCNB1* cyclin B1; *CDK* cyclin-dependent kinase; *DNMT1* DNA methyltransferase 1; *dsRNA* double-stranded RNA; *ER* estrogen receptor; *ERV* endogenous retrovirus genes; *FOXM1* forkhead box protein M1 transcription factor; *IC*_*50*_ half maximal inhibitory concentration; *IFN* interferon; *NR* not reported; *M* mitosis; *MDM4* p53 regulator; *MEP50* methylosome protein 50; *mTOR* mammalian target of rapamycin; *P* phosphorylated; *p53* tumor protein 53; *PKL1* polo-like kinase 1; *PD-L1* programmed death-ligand 1; *PFK1* 6-phosphofructokinase; *PI3K* phosphatidylinositol-3-kinase; *PKM2* pyruvate kinase M2; *PRMT5* protein arginine methyltransferase 5; *Rb* retinoblastoma; *RTK* receptor tyrosine kinase; *SPOP* speckle-type POZ protein

Cyclin-dependent kinase 4/6 (CDK4/6) and cyclin D1 together play a role in regulating cell-cycle progression (Fig. 1) [8, 9]. Palbociclib was the first CDK4/6 inhibitor approved for the treatment of HR+/HER2– ABC in combination with an aromatase inhibitor in the first-line setting or fulvestrant in the second-line setting in the United States [10]. In Japan, palbociclib was approved for the treatment of inoperable or recurrent breast cancer in 2017 [11]. Current Japanese Breast Cancer Society Clinical Practice guidelines recommend a CDK4/6 inhibitor plus an aromatase inhibitor as a first-line endocrine therapy for postmenopausal patients with HR+/HER2‒ ABC [12].

Early preclinical studies showed that palbociclib was a potent and highly selective CDK4/6 inhibitor, and that targeting CDK4/6 alone resulted in antitumor activity [13, 14]. Two other CDK4/6 inhibitors, ribociclib and abemaciclib, have also been developed for the treatment of HR+/HER2– ABC [15]. The clinical benefits of palbociclib [16, 17], ribociclib [18, 19], and abemaciclib [20, 21] have been shown in randomized clinical trials, with all 3 CDK4/6 inhibitors showing comparable efficacy results and manageable safety profiles without a deterioration in quality of life [22]. The focus of this review is to summarize current clinical trial data and real-world evidence supporting palbociclib as the first-line standard of care for HR+/HER2– ABC.

## Review of palbociclib clinical trial data

### Palbociclib clinical trials

The phase 3 PALOMA-2 and PALOMA-3 trials showed significant improvement in PFS with palbociclib plus letrozole or fulvestrant versus placebo plus letrozole or fulvestrant, respectively (Table 1) [16, 17, 23–25]. In PALOMA-2, postmenopausal women with ER+/HER2– ABC were randomized 2:1 to receive palbociclib or placebo plus letrozole as first-line treatment for their advanced disease [16, 23]. The median PFS was 27.6 months in the palbociclib group versus 14.5 months in the placebo group. Furthermore, a subgroup analysis of patients enrolled in PALOMA-2 showed a PFS benefit with palbociclib plus letrozole versus placebo plus letrozole across all patient subgroups, including among patients with bone-only disease [23]. Moreover, median PFS was significantly longer in the palbociclib group compared with the placebo group among patients with visceral metastases (19.3 months [95% CI, 16.4–22.2] vs 12.9 months [8.4–16.6], respectively; hazard ratio = 0.63 [95% CI, 0.47–0.85]; *P* < 0.01), and among patients without visceral metastases (not reached [95% CI, 25.1–not estimable] vs 16.8 months [95% CI, 13.7–22.2]; hazard ratio = 0.50 [95% CI, 0.36–0.70]; *P* < 0.0001) [26]. In PALOMA-3, pre/perimenopausal and postmenopausal women with HR+/HER2– ABC whose cancer had relapsed or progressed with prior endocrine therapy were randomized 2:1 to receive palbociclib or placebo plus fulvestrant [17, 24]. Median PFS was 11.2 and 4.6 months in the palbociclib and placebo groups, respectively. Moreover, OS analysis from PALOMA-3 after 44.8 months of follow-up showed an OS benefit of 6.9 months with palbociclib plus fulvestrant versus placebo plus fulvestrant [25]. Overall survival data from PALOMA-2 have not yet been reported.

**Table 1:** Efficacy and safety outcomes in patients with HR+/HER2– ABC treated with palbociclib in clinical trials and real-world studies

Outcome	Clinical trials		Real-world studies^a^						
	PALOMA-2 overall population [16, 23] PAL + LET vs PBO + LET (First-line)	PALOMA-3 overall population [24, 25, 62] PAL + FUL vs PBO + FUL (Second-line)	Pizzuti et al. [48] PAL + AI or FUL	Taylor-Stoke et al. [46] PAL + AI or FUL	Waller et al. [47] PAL + LET or FUL	Varella et al. [49] PAL + ET	Wilkie et al. [50] PAL + AI	Watson et al. [52] PAL + ET	Xi et al. [51] PAL + ET
Country	Global	Global	Italy	United States	Argentina	United States	United States	Ireland	United States
Total number of patients	666 (444, PAL group; 222 PBO group)	521 (347 PAL group; 174 PBO group)	423	652	162	411	70	64	200
Prior chemotherapy for ABC	No	Yes (*n* = 107/347 in PAL group; *n* = 63/174 in PBO group)	Yes (*n* = 165)	Yes (*n* = 28/360 in PAL + AI group; *n* = 15/292 in PAL + FUL group)	Yes (*n* = 5/105 in PAL + LET group; *n* = 7/57 in PAL + FUL group)	NA	No	Yes (*n* = 21)	NA
Treatment line of palbociclib for ABC	1L	1L (24.2%), 2L (38.0%), 3L (25.9%), and ≥ 4L (11.8%)	1L (37.3%) and ≥ 2L (62.7%)	1L (57.7%), 2L (34.8%), and ≥ 3L (7.5%)	1L (65%), 2L (31%), and 3L (4%)	1L (35.8%), 2L (26.0%), 3L (12.9%), and ≥ 4L (25.3%)	1L	1L (40.6%) and ≥ 2L (59.4%)	1L (21.0%), 2L (25.0%), and ≥ 3L (54.0%)
Endocrine therapy	LET	FUL	AI or FUL	AI or FUL	LET or FUL	LET, FUL, exemestane, tamoxifen, or anastrozole	AI	LET, faslodex, exemestane, tamoxifen	LET, FUL, anastrozole, or tamoxifen
Menopausal status	Post	Peri/pre and post	Pre and post	Post	Pre and post	Pre and post	Post	Pre and post	Pre and post or male
Median PFS, mo (95% CI)	27.6 (22.4–30.3) vs 14.5 (12.3–17.1)	11.2 (9.5–12.9) vs 4.6 (3.5–5.6)	12.0 (8.0‒16.0)^d^	NA	NA	PAL + LET: 1L: 15.1 mo (12.3–not reached) PAL + FUL: 2L: 12.3 (8.7–not reached)	1L: 26.4 (19.7–33.2)	NA	1L: 20.7 2L: 12.8 ≥ 3L: 4.0
Hazard ratio (95% CI)	0.56 (0.46–0.69)	0.50 (0.40–0.62)	NA	NA	NA	NA	NA	NA	NA
* P* value	< 0.0001	< 0.0001	NA	NA	NA	NA	NA	NA	NA
Median OS, mo (95% CI)	NA	34.9 (28.0‒40.0) vs 28.0 (23.6‒34.6)	24.0 (17.0‒30.0)^d^	NA	NA	PAL + LET: NR^d^ PAL + FUL: 24.5^d^	NA	NA	NA
Hazard ratio (95% CI)	NA	0.81 (0.64‒1.03)	NA	NA	NA	NA	NA	NA	NA
* P* value	NA	0.09	NA	NA	NA	NA	NA	NA	NA
OR rate, % (95% CI)	55.3 (49.9–60.7) vs 44.4 (36.9–52.2)^b^	25.0 (19.6–30.2) vs 11.0 (6.2–17.3)^b^	31.0 (26.6‒35.4)^d^	77.1	66	NA	NA	NA	NA
Odds ratio (95% CI)	1.55 (1.05–2.28)	2.69 (1.43–5.26)	NA	NA	NA	NA	NA	NA	NA
*P* value	0.03	0.0012	NA	NA	NA	NA	NA	NA	NA
CBR rate, % (95% CI)	84.3 (80.0–88.0) vs 70.8 (63.3–77.5)^b^	64.0 (57.7–69.6) vs 36.0 (28.2–44.8)^b^	52.7 (48.0‒57.5)^d^	90.0‒93.6	87‒94	NA	NA	NA	NA
Odds ratio (95% CI)	2.23 (1.39–3.56)	3.10 (1.99–4.92)	NA	NA	NA	NA	NA	NA	NA
*P* value	< 0.001	< 0.0001	NA	NA	NA	NA	NA	NA	NA
Most frequent AEs, %	Neutropenia (79.5 vs 6.3) Leukopenia (39.0 vs 2.3) Fatigue (37.4 vs 27.5)	Neutropenia (80.9 vs 3.5) Infections (41.7 vs 30.2) Fatigue (39.1 vs 28.5)	Neutropenia Anemia Fatigue	NA	NA	Hematologic AEs Fatigue	NA	Neutropenia (95.3)	NA
Grade 3 or 4 neutropenia, %	66.4 vs 1.4	64.6 vs 1.0	37.1 (grade 3) 6.1 (grade 4)	NA	NA	57.7	62	NA	38.5 (grade 3) 3.0 (grade 4)
Subsequent therapies	Median time to initiation of first subsequent therapy was 28.0 mo with PAL + LET vs 17.7 months with PBO + LET Time to second subsequent therapy was 38.8 mo with PAL + LET vs 28.8 mo with PBO + LET Among patients who permanently discontinued treatment, ET was the most common first subsequent treatment Median time to first-line subsequent chemotherapy was 40.4 mo with PAL + LET vs 29.9 mo with PBO + LET	40% received endocrine-based therapy Time to first subsequent chemotherapy was 17.6 mo with PAL + FUL and 8.8 mo with PBO + FUL	NA	NA	NA	NA	NA	NA	67.3% received chemotherapy, 30.8% hormone therapy
QoL	Overall change from baseline in FACT-B total scores was not significantly different between PAL + LET and PBO + LET	Mean overall change from baseline in EORTC QLQ-C30 score^c^ (–0.9 points with PAL + FUL vs –4.0 points with PBO + FUL)	NA	NA	NA	NA	NA	NA	NA

*1L* first-line; *2L* second-line; *3L* third-line; *ABC* advanced breast cancer; *AE* adverse event; *AI* aromatase inhibitor; *CBR* clinical benefit response; *EORTC* European Organisation for Research and Treatment of Cancer; *ET* endocrine therapy; *FACT-B* Functional Assessment of Cancer Therapy-Breast; *FUL* fulvestrant; *HER2* human epidermal growth factor receptor 2; *HR* hormone receptor; *LET* letrozole; *NA* not available; *NE* not estimable; *OR* objective response; *OS* overall survival; *PAL* palbociclib; *PBO* placebo; *PFS* progression-free survival; *QLQ-C30* Quality of Life Core Module; *QoL* quality of life

^a^Included real-world studies with > 50 patients

^b^Among patients with measurable disease

^**c**^Higher scores indicate a higher QoL (range, 0–100)

^d^All lines of therapy combined

In both phase 3 PALOMA trials, neutropenia was the most common grade 3 or 4 adverse event (AE) [16, 17]. Among patients in the palbociclib plus letrozole group in PALOMA-2, 56.1% experienced grade 3 neutropenia and 10.4% experienced grade 4 neutropenia; the incidence of grade 3 or 4 febrile neutropenia was low (1.8%) [16]. In PALOMA-3, among patients in the palbociclib plus fulvestrant group, 53.3% experienced grade 3 and 8.7% experienced grade 4 neutropenia; febrile neutropenia was reported in 0.6% of patients [17]. A pooled analysis of data from the PALOMA-1, -2, and -3 trials showed that interstitial lung disease was reported in 1.5% of patients receiving palbociclib plus endocrine therapy and that the incidence of interstitial lung disease was similar across geographic locations [27]. Additionally, quality of life was maintained in patients treated with palbociclib plus endocrine therapy [28, 29].

Subsequent treatments after permanent discontinuation of palbociclib were also assessed in both phase 3 trials **(**Table 1). These findings indicate that palbociclib did not compromise the efficacy of subsequent therapy, and that palbociclib combination therapy extended the time to chemotherapy [23, 25]. Moreover, the types of subsequent therapies patients received were generally similar between treatment arms in both PALOMA-2 and PALOMA-3, suggesting that palbociclib does not influence the subsequent therapy received [23, 25].

**Fig. 2:**
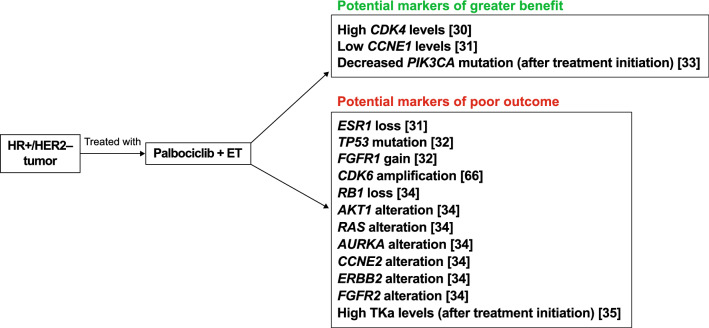
Potential biomarkers predictive of response to palbociclib [30–35, 66]. These markers have the potential to predict response to palbociclib in patients with HR+/HER2– breast cancer. *ER* estrogen receptor; *ET* endocrine therapy; *HER2*– human epidermal growth factor receptor 2–negative; *HR*+hormone receptor–positive; *PIK3CA* phosphatidylinositol-4,5-bisphosphate 3-kinase catalytic subunit alpha; *TKa* thymidine kinase 1 activity

Biomarker analyses using patient tumor samples were conducted in both PALOMA-2 and PALOMA-3. Data from PALOMA-2 reinforced ER status as a significant marker for therapeutic benefit with observations supporting that this breast cancer subtype is dependent on the CDK4/6:cyclin D:retinoblastoma pathway [30]. In addition, higher levels of *CDK4* gene expression were suggestive of an endocrine resistance phenotype that could be circumvented with the addition of palbociclib (Fig. 2) [30]. Analyses of PALOMA-3 showed that lower levels of *CCNE1* mRNA expression were linked to greater PFS benefit with palbociclib plus fulvestrant treatment [31]. Analyses of circulating tumor DNA (ctDNA) from PALOMA-3 showed that with both palbociclib plus fulvestrant and placebo plus fulvestrant treatment, *TP53* mutation and *FGFR1* gain were associated with early relapse [32]. Moreover, *PIK3CA* ctDNA dynamics after 2 weeks of palbociclib plus fulvestrant treatment were predictive of long-term outcomes [33].

Additionally, an analysis was performed based on whole-exome sequencing of 59 tumors from patients with HR+/HER2‒ MBC who received CDK4/6 inhibitors to evaluate mechanisms driving resistance to CDK4/6 inhibitors [34]. The study identified loss of *RB1*; and alterations in *AKT1*, *RAS*, *AURKA*, *CCNE2*, *ERBB2*, and *FGFR2* as potential CDK4/6 inhibitor resistance mechanisms [34]. A study also showed that an increase in plasma levels of thymidine kinase 1 activity (TKa) after 1 cycle of palbociclib treatment was associated with shorter median PFS, highlighting TKa levels as a predictor of early resistance to CDK4/6 inhibition [35]. Further research is warranted to confirm whether previously identified potential genomic biomarkers are predictive of response to CDK4/6 inhibitors [36].

### Palbociclib clinical trial data in Asian and Japanese patients

Subgroup analyses of Asian patients enrolled in PALOMA-2 and PALOMA-3 showed a favorable efficacy and pharmacokinetic and safety profile generally consistent with that of the overall population. Ninety-five Asian patients (14.3% of the overall population), including 46 Japanese patients, were enrolled in PALOMA-2, and 105 Asian patients (20.2% of the overall population), including 35 Japanese patients, were enrolled in PALOMA-3 [37–39]. Among Asian patients in PALOMA-2, median PFS was 25.7 months with palbociclib plus letrozole versus 13.9 months with placebo plus letrozole (hazard ratio, 0.49 [95% CI, 0.27–0.87]; *P* = 0.007) [37]. Compared with non-Asians, Asians had higher geometric mean palbociclib *C*_trough_ values (93.8 vs 61.7 ng/mL), but variability was lower (percent coefficient of variance: 32.3% vs 59.1%), and the distribution of *C*_trough_ values was generally similar [37]. In PALOMA-3, among Asian patients, median PFS was not reached in the palbociclib group and was 5.8 months in the placebo group (hazard ratio, 0.485 [95% CI, 0.27–0.87]; *P* = 0.0065) [39]. Asian and non-Asian patients had similar palbociclib exposure, with similar within-patient geometric mean steady-state palbociclib trough concentration values (85.7 and 74.8 ng/mL, respectively) [39]. In both studies, neutropenia was the most common any-grade AE among Asian patients who received palbociclib, and was also more frequent among Asian than non-Asian patients [37, 39]. However, palbociclib discontinuation rates due to AEs among Asian and non-Asian patients were comparable (PALOMA-2: 10.8% vs 9.5%; PALOMA-3: 0% vs 5.1%), suggesting that palbociclib AEs are manageable in Asian patients.

Subgroup analyses specifically looking at the Japanese cohorts from PALOMA-2 and PALOMA-3 were also conducted (Table 2). A total of 46 Japanese patients with ER+/HER2– ABC were enrolled in PALOMA-2; 32 patients received palbociclib plus letrozole and 14 received placebo plus letrozole [38]. Median PFS among Japanese patients was longer with palbociclib compared with placebo (22.2 vs 13.8 months; *P* = 0.103), and PFS was not affected by dose reduction [38]. At the time of this report, OS data were not yet mature. Compared with that in non-Asians, the steady-state geometric mean palbociclib *C*_trough_ value was higher in Japanese patients (95.4 vs 61.7 ng/mL), but the individual values were within a similar range. The incidence of any-grade hematologic AEs observed in the palbociclib group was higher among Japanese patients than that observed in the overall population of PALOMA-2 (Table 2). Overall, grade 3/4 AEs were observed more frequently among Japanese patients treated with palbociclib than those in the overall population. For example, neutropenia was observed in 87.5% of palbociclib-treated Japanese patients versus 66.4% of patients in the overall population. More Japanese patients experienced a dose reduction due to an AE compared with the overall population; neutropenia was associated with dose reduction in 31.3% of patients [38]. Overall, neutropenia was manageable with dose modification, and only 9.4% of patients discontinued study treatment because of neutropenia.

**Table 2:** Efficacy and safety outcomes in Japanese patients with HR+/HER2– ABC treated with palbociclib in clinical trials and real-world studies

Outcome	Clinical trials			Real-world studies			
	PALOMA-2 Japanese subgroup analysis [38, 45] PAL + LET vs PBO + LET	PALOMA-3 Japanese subgroup analysis [40, 45] PAL + FUL vs PBO + FUL	Japanese phase 2 single-arm study [43, 44] PAL + LET	Seki et al. 2019 [55] PAL + FUL vs FUL	Iwatomo et al. 2018 [56] PAL + AI or FUL	Kikuchi et al. 2019 [57] PAL + AI or FUL	Odan et al. 2020 [58] PAL + AI, SERD, or SERM
Total number of Japanese patients	46 (32, PAL group; 14 PBO group)	35 (27 PAL group; 8 PBO group)	42	70 (31 PAL group; 39 FUL group)	26	35	177
Endocrine therapy	LET	FUL	LET	FUL	AI or FUL	AI or FUL	AI, SERD, or SERM
Menopausal status	Post	Peri/pre and post	Post	Peri/pre and post	Pre/post	NA	Peri/pre and post
Prior chemotherapy for ABC	No	Yes (*n* = 2/27 in PAL group; *n* = 1/8 in PBO group)	No	Yes	Yes (*n* = 11/26)	NA	Yes (*n* = 97)
Treatment line of palbociclib for ABC	1L	1L (25.9%), 2L (44.4%), 3L (18.5%), and ≥ 4L (11.1%)	1L	1L (9.7%), 2L (35.5%), 3L (16.1%), and ≥ 4L (38.7%)	1L (8%), ≥ 4L (46%), and ≥ 5L (46%)	1L (22.9%), 2L (8.6%), 3L (20.0%), 4L (28.6%), and ≥ 5L (20.0%)	1L (11%), 2L (15%), and 3L (73%)
Median PFS, mo (95% CI)	22.2 (13.6–NE) vs 13.8 (5.6–22.2)	13.6 (7.5–NE) vs 11.2 (5.6–NE)	35.7 (21.7–46.7)	13.3 vs 3.9	NR (upfront-line^a^) 3.6 (later-line^a^)	NA	NA
Hazard ratio (95% CI)	0.59 (0.26–1.34)	0.82 (0.32–2.11)	NA	0.272 (0.128–0.574)	NA	NA	NA
*P* value	0.103	0.339	NA	< 0.001	NA	NA	NA
OR rate, % (95% CI)	46.4 (27.5–66.1) vs 38.5 (13.9–68.4)	23.8 (8.2–47.2) vs 25.0 (3.2–65.1)	55.6 (38.1–72.1)	2.6 vs 41.9	NA	17	NA
Odds ratio (95% CI)	1.39 (0.30–6.79)	0.94 (0.11–12.41)	NA	NA	NA	NA	NA
*P* value	0.4465	0.7177	NA	< 0.001	NA	NA	NA
CBR rate, % (95% CI)	75.0 (55.1–89.3) vs 84.6 (54.6–98.1)	71.4 (47.8–88.7) vs 87.5 (47.3–99.7)	83.3 (67.2–93.6)	23.1 vs 61.3	NA	71.4	NA
Odds ratio (95% CI)	0.55 (0.05–3.63)	0.36 (0.007–4.07)	NA	NA	NA	NA	NA
*P* value	0.8650	0.9255	NA	0.002	NA	NA	NA
Most frequent AEs, %	Neutropenia (93.8 vs 14.3) Leukopenia (62.5 vs 7.1) Stomatitis (53.1 vs 28.6)	Neutropenia (93.0 vs 25.0) Leukopenia (74.0 vs 13.0) Stomatitis (44.0 vs 25.0)	Neutropenia (100.0) Leukopenia (83.3) Stomatitis (76.2)	Leukopenia Neutropenia Anemia Fatigue	Neutropenia (100.0, upfront- and later-line) Thrombocytopenia (50.0, upfront-line; 33.0, later-line) Anemia (71.0, upfront-line; 50.0, later-line)	Leukopenia (69) Neutropenia (74) Anemia (37)	Neutropenia ( 92.7), Leukopenia (92.1), Anemia (60.5), Thrombocytopenia (52.5), Elevation of liver enzymes (21.5)
Grade 3 or 4 neutropenia, %	87.5 vs 0	92.6 vs 0	92.9	80.6	85.7 (upfront-line) 83.3 (later-line)	46	71.2
Subsequent therapies	PAL + LET: 69% received first subsequent therapy (77% ET, 18% chemotherapy) PBO + LET: 86% received first subsequent therapy (75% ET, 8% chemotherapy)	PAL + FUL: 81% received first subsequent therapy (55% ET, 32% chemotherapy) PBO + FUL: 88% received first subsequent therapy (43% ET, 57% chemotherapy)	54.8% received subsequent systemic therapies (87% ET, 13% chemotherapy)	NA	NA	NA	NA

*ABC* advanced breast cancer; *AE* adverse event; *AI* aromatase inhibitor; *CBR* clinical benefit response; *ET* endocrine therapy; *FUL* fulvestrant; *HER2* human epidermal growth factor receptor 2; *HR* hormone receptor; *LET* letrozole; *NA* not available; *NE* not estimable; *OR* objective response; *PAL* palbociclib; *PBO* placebo; *PFS* progression-free survival; *SERD* selective estrogen receptor degrader; *SERM* selective estrogen receptor modulator

^a^Upfront line was defined as patients with ≤ 3 prior lines of therapy; later-line was defined as patients with ≥ 4 prior lines of therapy

PALOMA-3 enrolled 35 Japanese patients with HR+/HER2– MBC whose disease had progressed on previous endocrine therapy; 27 patients received palbociclib plus fulvestrant and 8 patients received placebo plus fulvestrant [40]. Median PFS was longer among Japanese patients who received palbociclib versus those who received placebo (13.6 vs 11.2 months; *P* = 0.339). Japanese and non-Asian patients had similar within-patient geometric mean *C*_trough_ palbociclib levels at steady state (84.4 and 74.8 ng/mL, respectively), showing similar palbociclib exposure. Similar to the overall population, neutropenia was the most common AE with palbociclib treatment among Japanese patients; albeit, a higher rate of neutropenia was observed in Japanese patients versus the overall population (93.0% vs 79.0%). Febrile neutropenia was reported in 1 Japanese patient receiving palbociclib plus fulvestrant. Although more Japanese patients experienced a palbociclib dose reduction due to hematologic AEs (33% due to neutropenia) than in the overall population, no Japanese patient discontinued palbociclib treatment because of AEs.

In a pooled analysis of Japanese patients from PALOMA-2, PALOMA-3, and the single-arm Japanese phase 2 study (*n* = 101), 98.0% of Japanese patients who received palbociclib experienced all-grade neutropenia [41]; grade 3 or 4 neutropenia was reported in 90.1% of patients, but was manageable with dose modifications. Compared with Japanese patients who required cycle delay or dose interruption, patients who completed a 3/1 schedule during the first 2 cycles with palbociclib had higher baseline neutrophil counts. In PALOMA-2 and the Japanese phase 2 study, baseline neutrophil levels were positively correlated with neutrophil count at Cycle 1 Day 15. Importantly, exposure–response analyses in the overall populations in PALOMA-2 and PALOMA-3 showed similar PFS in patients with and without palbociclib dose reductions [42], and palbociclib dose reduction did not affect tumor response in Japanese patients [41]. In addition, no apparent correlation was observed between the post-treatment absolute neutrophil count and *C*_trough_ in this pooled analysis or in PALOMA-2 or PALOMA-3, suggesting that the pharmacokinetics of palbociclib do not affect the incidence of neutropenia [38, 40, 41].

An open-label, single-arm, Japanese phase 2 study also examined the efficacy of palbociclib plus letrozole among postmenopausal patients with ER+/HER2– ABC who had no prior systemic anticancer therapy for ABC [43, 44]. A total of 42 patients received palbociclib plus letrozole and were included in efficacy analyses. In an updated analysis of the primary study [44], the 1-year PFS probability was 75.6%, and the median PFS was 35.7 months. All 42 Japanese patients experienced any-grade neutropenia. Grade 3 or 4 neutropenia was observed in 92.9% of patients, and treatment-related febrile neutropenia (grade 3) was observed in 1 patient. In general, palbociclib-related AEs observed in the Japanese phase 2 study were managed through dose modification without affecting treatment duration or efficacy. Among the 23 patients (54.8%) who received ≥ 1 subsequent anticancer therapy, the majority received endocrine therapy (87.0%) followed by chemotherapy (13.0%). Similar to the Japanese phase 2 study, a recent report on subsequent treatment patterns after palbociclib plus endocrine therapy or placebo plus endocrine therapy in Japanese patients enrolled in PALOMA-2 and PALOMA-3 showed that endocrine therapy was the most common first subsequent therapy; chemotherapy was the second most comment subsequent therapy [45].

## Treatment with palbociclib in the real-world setting

### Real-world evidence regarding treatment with palbociclib

Palbociclib treatment in the real-world setting has been assessed in several retrospective studies and further support the efficacy and safety of palbociclib plus endocrine therapy for HR+/HER2– ABC. Table 1 summarizes real-world data from palbociclib studies that included more than 50 patients who were pre/peri- or postmenopausal women or men with HR+/HER2– ABC. The Ibrance Real World Insights (IRIS) study used medical chart review data to evaluate palbociclib treatment in patients with confirmed HR+/HER2– ABC who received palbociclib in combination with either an aromatase inhibitor or fulvestrant in the United States, Argentina, and Germany [46, 47]. In addition, other real-world studies have published data from the United States, Italy, and Ireland [48–52]. In summary, real-world PFS was 15.1–26.4 months in the first-line setting [49–51], and 12.3–12.8 months in the second-line setting [49, 51], indicating that real-world efficacy with palbociclib combination treatment complements that observed in randomized controlled trials (PFS: PALOMA-2, 27.6 months in the first-line setting [23]; PALOMA-3, 11.2 months in the second- or later-line setting [24]). Similar to PALOMA-2 and PALOMA-3, commonly reported AEs in real-world studies included neutropenia, other hematologic AEs, and fatigue [48, 49, 52]. Of note, interstitial lung disease was also observed with CDK4/6 inhibitors in the real-world setting as well as PALOMA studies [27, 53]. Additionally, real-world data were utilized to expand the approved indications of palbociclib to include male patients with ABC in 2019 [54].

In Japan, the real-world efficacy of palbociclib was analyzed in 4 retrospective studies (Table 2) [55–58]. Whereas the sample sizes of Japanese real-world studies were small, the efficacy and safety results seem to be consistent with global real-world data. Findings from clinical trials and real-world data in Japanese patients showed that AEs associated with palbociclib therapy, including neutropenia, are managed effectively by dose modifications. However, further studies are needed to confirm the clinical effect of palbociclib in Japan, such as on efficacy, survival, AEs, and cost-effectiveness.

## Discussion

CDK4/6 inhibitors have been shown to prolong PFS and OS as first-line or second-line treatment in patients with ABC [19, 20, 23–25, 59, 60]. Several reports in both the clinical trial and real-world settings have shown that the magnitude of PFS benefit is greater when palbociclib is used as an early-line therapy rather than in later-line settings, suggesting a limited clinical benefit among patients who receive it as a later-line option. First, hormone sensitivity decreases with subsequent endocrine therapy treatments, resulting in a reduced clinical benefit rate [61]. Findings from PALOMA-3 showed that among patients with sensitivity to previous endocrine therapy, median OS was 10 months longer with palbociclib plus fulvestrant versus placebo plus fulvestrant (hazard ratio for death, 0.72) [25]. Second, time to first subsequent chemotherapy with palbociclib treatment in PALOMA-2 was longer than that observed in PALOMA-3 (40.4 vs 17.6 months), suggesting quality of life was maintained for a longer period of time before chemotherapy was initiated [23, 25]. Finally, objective response with palbociclib treatment in PALOMA-2 was higher than that in PALOMA-3 among patients with measurable disease (55.3% vs 25.0%) [16, 62]. It has also been shown that first-line treatment response was a key predictor of post-recurrence survival in patients with HR+/HER2– breast cancer [63]. Poor responses to first-line treatment were associated with unfavorable prognostic outcomes [63]. Success of first-line treatment may result in a positive and long-term relationship between doctor and patient. Together, these results suggest that in patients with ABC or MBC, the optimal treatment option should be prescribed first-line.

Elucidating biomarkers that are predictive of palbociclib treatment benefit may highlight the optimal clinical application of this CDK4/6 inhibitor in patients with ABC, including in those who are endocrine therapy–naive. Current evidence suggests that plasma thymidine kinase activity may predict response to palbociclib [35, 64]. Biomarkers such as this will be especially helpful to identify patients who will derive the greatest benefit from palbociclib combination therapy, including identifying patients sensitive to endocrine therapy who derive greater benefit from palbociclib. Moreover, it will be important to determine if such biomarkers are also predictive of treatment benefit in Asian and Japanese patients. In the event biomarkers identified in the overall population are not predictive in Asian patients, additional biomarker analyses in this population will be warranted.

Although AEs (e.g., neutropenia) are the main cause of palbociclib dose modification, analyses have suggested that there is no difference in efficacy between patients who did or did not experience a dose reduction [41]. Additionally, exposure–response analyses have shown that palbociclib dose reductions do not affect PFS [42]. Thus, AEs can be managed via dose modification without affecting the PFS benefit provided by palbociclib combination therapy.

Currently, several clinical trials of palbociclib for HR+/HER2– ABC are ongoing to address remaining clinical questions. First, it is not clear whether the optimal benefit of palbociclib can be achieved through first- or second-line treatment. The SONIA trial (ClinicalTrials.gov Identifier: NCT03425838) is currently evaluating whether the sequence of an aromatase inhibitor plus CDK4/6 inhibitor as first-line therapy, followed by fulvestrant as second-line therapy, is more effective than an aromatase inhibitor as first-line therapy followed by fulvestrant plus a CDK4/6 inhibitor as second-line therapy. Second, additional research on the choice of endocrine partner for CDK4/6 inhibitor combination therapy is also needed, as some clinical studies have already investigated the treatment benefit of using tamoxifen or an aromatase inhibitor with ribociclib [65]. Moreover, novel oral selective estrogen receptor degraders in combination with palbociclib are currently being evaluated (ClinicalTrials.gov Identifier: NCT03455270, NCT04711252).

Additionally, evaluation of the choice of subsequent therapy after disease progression while receiving palbociclib or after discontinuation of palbociclib due to an AE is warranted. The clinical study MAINTAIN is currently assessing the efficacy of ribociclib in patients whose disease progressed while receiving a CDK4/6 inhibitor (ClinicalTrials.gov Identifier: NCT02632045). A further understanding of the mechanisms of resistance to CDK4/6 inhibitors would aid in the assessment of subsequent treatment patterns. Current evidence suggests that the upregulation of various genes, such as CDK6 or *CCNE1*, may lead to resistance [66, 67]. Furthermore, real-time monitoring of tumor biology by ctDNA, as was demonstrated in PALOMA-3 [32, 33], may be a reasonable option for selecting optimal therapy depending on tumor characteristics (e.g., detection of the *PIK3CA* mutation would result in the selection of a PI3K inhibitor, such as buparlis or alpelisib [68]). The PADA-1 trial (ClinicalTrials.gov Identifier: NCT03079011), which is monitoring ctDNA for the occurrence of an *ESR1* mutation in patients with ER+/HER2– MBC receiving palbociclib plus an aromatase inhibitor, may also be helpful in identifying the optimal subsequent therapy [69]. This type of personalized medicine is expected in the near future.

Finally, it is essential to understand which patients will achieve an OS benefit from a CDK4/6 inhibitor. For instance, there are three types of patients that can be identified via the Kaplan–Meier plots of PALOMA-2 and PALOMA-3 studies: (1) an early resistance group who have disease progression within approximately 6 months of treatment, (2) patients who experience disease progression near the median PFS time, and (3) patients who achieve a PFS benefit longer than the median PFS [16, 17, 23, 24]. Thus far, there is minimal evidence to identify which patients will have longer survival with CDK4/6 inhibitor treatment, highlighting the importance of identifying a biomarker for this population. In addition, strategies to extend OS in patient groups 1 and 2 mentioned previously are warranted.

As was reviewed in this manuscript, several real-world studies demonstrate the efficacy and safety of palbociclib in clinical practice; however, there is still a lack of information available to answer the clinical questions discussed. Additional prospective clinical research studies and translational research studies are essential to help clarify these clinical questions associated with CDK4/6 inhibitor treatment.

## Conclusion

Early-line palbociclib treatment of patients with HR+/HER2– ABC provides clinical benefit regardless of patient ethnicity. Many clinical trial and real-world studies have highlighted the prolonged PFS afforded by palbociclib combination therapy compared with endocrine therapy alone when used as a first-line treatment in patients with HR+/HER2– ABC, in the overall population and in subgroups of Asian and Japanese patients. The safety profile of palbociclib therapy, especially neutropenia, is manageable through dose modification without affecting treatment duration or efficacy both in clinical trials and real-world studies. Analyses suggest potential biomarkers could be predictive of response to CDK4/6 inhibitors (e.g., *CDK4, CCNE1* levels). Further clinical research on biomarkers is merited to help improve outcomes in patients with HR+/HER2– ABC treated with CDK4/6 inhibitors as personalized medicine.
